# Integrative multi-omics and preclinical analyses identify miR-4776-5p as a prognostic radiosensitizer for patients undergoing radiotherapy for head-and-neck cancer

**DOI:** 10.1007/s12672-025-03784-6

**Published:** 2025-10-28

**Authors:** Yo-Liang Lai, Chun-Chieh Wang, Kai-Wen Hsu, Ching-Fang Yu, Yung-Lun Lin, Pei-Chun Shen, Meng-Hsin Tsai, Fang-Hsin Chen, Wei-Chung Cheng

**Affiliations:** 1 Department of Radiation Oncology, China Medical University Hospital, Taichung, Taiwan; 2 School of Medicine, College of Medicine, China Medical University, Taichung, Taiwan; 3 Department of Radiation Oncology, Chang Gung Memorial Hospital Linkou Branch, Taoyuan, Taiwan; 4 Drug Development Center, Program for Cancer Biology and Drug Discovery, China Medical University, Taichung, Taiwan; 5 Institute of Translational Medicine and New Drug Development, China Medical University, Taichung, Taiwan; 6 Institute for Radiological Research, Chang Gung University, Taoyuan, Taiwan; 7 Cancer Biology and Precision Therapeutics Center, China Medical University, Taichung, Taiwan; 8 Institute of Nuclear Engineering and Science, National Tsing Hua University, Hsinchu, Taiwan; 9 Program for Cancer Biology and Drug Discovery, China Medical University and Academia Sinica, Taichung, Taiwan

**Keywords:** Bioinformatics, miRNA, Radiotherapy, Cancer

## Abstract

Radiotherapy remains a cornerstone treatment for head-and-neck cancer (HNC), yet resistance to radiation therapy significantly limits clinical outcomes. MicroRNAs (miRNAs) have emerged as promising regulators of radiosensitivity; however, the mechanisms by which miRNAs modulate radiosensitivity in HNC remain incompletely understood. In this study, we employed a multi-omics strategy integrating transcriptomic, clinical, and outcome data to investigate miRNA-associated radiosensitivity. Critically, we defined two distinct patient cohorts in the public dataset: one group received only radiotherapy, and the other did not receive radiotherapy. This stratification allowed us to conduct a unique comparative bioinformatics analysis, leading to the identification of miR-4776-5p as a candidate radiosensitizer. Elevated miR-4776-5p expression correlated with improved prognosis, specifically in patients receiving radiotherapy. Functional assays confirmed that miR-4776-5p sensitized FaDu HNC cells to radiation-induced DNA damage and impaired clonogenic survival. Mechanistic analyses demonstrated that miR-4776-5p modulates the cell cycle and DNA damage response pathways, enhancing tumor radiosensitivity. Furthermore, xenograft mouse models validated the radiosensitizing effects of miR-4776-5p in vivo, evidenced by significantly delayed tumor growth following irradiation. These findings highlight miR-4776-5p as a potential biomarker and therapeutic agent to improve radiotherapy efficacy in HNC, supporting further clinical exploration.

**Supplementary Information:**

The online version contains supplementary material available at 10.1007/s12672-025-03784-6.

## Introduction

Cancers arising from the mucosal lining of the oral cavity, pharynx, and larynx are collectively known as head and neck cancers (HNCs). HNC is the seventh most common cancer worldwide, with more than 660,000 cases and 320,000 deaths annually [28]. Among HNCs, 90% are squamous cell carcinomas (HNSCs) and the rest are adenocarcinomas. The current standard treatments for HNSC include surgery, radiotherapy, chemotherapy, targeted therapy, and immunotherapy. While outcomes are improving, they remain unsatisfactory. Radiotherapy can be either a definitive treatment or an adjuvant therapy. Postoperative adjuvant radiotherapy has been shown to increase the five-year cancer-specific and overall survival of patients with lymph node-positive HNSC compared to surgery alone [16]. Patients with specific risk factors, such as extracapsular extension, perineural infiltration, vascular embolism, and lymphatic invasion, are suggested for postoperative radiotherapy or concurrent postoperative radiotherapy and chemotherapy to achieve better outcomes [3]. However, the cancers of some patients are prone to progression after this type of intensive treatment. The failure of treatment after radiotherapy is correlated with the radioresistance of tumor cells. Therefore, a biomarker, which acts as a potential radiosensitizer, is needed to select patients who respond poorly to radiotherapy.

MicroRNAs (miRNAs) are endogenous RNAs, 19–23 nucleotides long, which play important regulatory roles by targeting messenger RNAs for posttranslational modification. miRNAs effectively suppress multiple target genes through imperfect sequence bases, with complementarity between miRNAs and their targets [2]. Abnormal miRNA expression is found in various tumors and contributes to tumor development, tumor progression, and events leading to treatment resistance [21]. The deregulation of miRNAs in tumors can be targeted by potential therapeutics either using miRNA replacement therapy with miRNA mimics or inhibiting miRNA function with anti-miRNAs [27]. For example, lipid nanoparticle-encapsulated miRNA-34 mimics showed promising anti-tumor activity when given to animal tumor models of prostate [17], liver [11], and lung [29] cancers and are currently being tested against several solid and hematological malignancies in a phase I clinical trial (NCT01829971) [27].

Several studies have reported that miRNA modulates the tumor response to ionizing radiation [9, 23, 26], and the growing evidence focuses on HNCs [22]. Most miRNAs were identified using microarray methods, and few predicted the prognosis of clinical patients. While some studies identified miRNAs using high-throughput screening with large sample sizes, they lacked supporting evidence from in vitro and in vivo studies [4, 13]. In this study, we identified an miRNA, miR-4776-5p, related to the clinical outcome of patients with HNSC receiving only radiotherapy from The Cancer Genome Atlas (TCGA) database and validated it using in vitro and in vivo experiments. This miRNA has the potential to be selected as a therapeutic target for blocking radioresistant characteristics, which could lead to better treatment outcomes for HNC.

## Materials and methods

### Data collection and processing

The miRNA and RNA expression profiles, along with clinical information for the TCGA-HNSC dataset, were obtained from our previous studies using the YM500v3 [5] and DriverDBv3 [20] databases. The miRNA-seq data were downloaded from CGHub (https://cghub.ucsc.edu/) and processed using the miRNA-seq pipeline provided by YM500. miRNA annotation was performed using the miRBase database R22 [14]. In addition, processed RNA-seq data, including coding genes, were also sourced from CGHub for further analysis. In total, this study curated 20,495 genes, 2588 miRNAs, and corresponding clinical data for comprehensive analysis.

### Differential expression and survival analyses

We performed differential expression analysis to identify miRNAs significantly differentially expressed between primary tumor and adjacent normal tissue samples using the R package DESeq (version 1.28.0) [5]. Differentially expressed miRNAs were selected based on an adjusted p-value of < 0.05 and a log2 fold change of > 3. To ensure data quality, miRNA candidates with normalized mean counts (baseMean) of < 1 were filtered out to exclude those with excessively low expression levels. In addition, we performed survival analysis to identify clinically relevant miRNA candidates. Patients were stratified into high and low expression groups based on the median miRNA expression. Cox proportional hazards models were then calculated using the R package survival (version 2.41–3) to estimate survival differences between these groups. miRNAs associated with clinical outcomes were identified based on a significance criterion of p < 0.05.

### miRNA function prediction

We predicted the functions of the miRNAs identified by analyzing their target genes. The target gene prediction methods were based on our previous work [5, 6]. Specifically, we employed 12 additional bioinformatics tools to predict interactions between miRNAs and their corresponding target genes. To further refine these predictions, we assessed the correlations between predicted target genes and miRNA expression levels using Spearman correlation analyses. Only miRNA–gene pairs showing significant negative correlations and predicted by at least one tool were considered for further analysis (Table S1). Finally, functional enrichment analysis of the target genes was performed using in-house scripts, as described in our previous study [7, 18, 20].

### miR-4776-5p mimics transfection and irradiation of FaDu cells

FaDu cells (American Type Culture Collection: HTB-43™) were cultured in minimal essential medium supplemented with 10% fetal bovine serum and 1% antibiotics and maintained at 37 °C in a humidified atmosphere with 5% carbon dioxide. For the experiments, cells were transfected with selected miR-4776-5p mimics or negative control (NC) mimics (mirVana miRNA mimics; Thermo Fisher Scientific, Waltham, MA, USA) at a final concentration of 40 nM using Lipofectamine 3000 (Invitrogen, Thermo Fisher Scientific, Waltham, MA, USA.), according to the manufacturer’s instructions. After transfection, cells were maintained until the designated study time points. The cells were irradiated with 6-MV X-rays from a linear accelerator (Clinac iX; Varian Medical System, Palo Alto, CA, USA.) at a dose rate of 6 Gy/min.

### Quantitative real-time PCR

After transfection with synthetic mimics (Negative Control [mirVana™, Cat No. 4464058] and hsa-miR-4776-5p mimic [sequence: GUGGACCAGGAUGGCAAGGGCU], mirVana^®^ miRNA mimic, Cat No. 4464066), total RNA was extracted using Trizol reagent at 24 h. miRNAs were reverse transcribed with the miRCURY LNA RT kit (QIAGEN), and mRNAs with the High-Capacity cDNA Reverse Transcription Kit (Applied Biosystems). qRT-PCR was performed on a CFX Connect™ system (Bio-Rad) using SYBR Green chemistry. miR-4776-5p expression was normalized to RNU6, while mRNA expression of SH2D2A, FBXO44, VKORC1, MCTS1, AP1M1, and PARVB was normalized to ACTB. Relative expression levels were calculated using the 2 − ΔΔCt method. Primer sequences are listed in Table S2.

### Colony formation assay

The transfected FaDu cells were seeded according to the irradiation dose. At higher doses, 2–3 times more cells were seeded compared to the non-irradiated control to ensure sufficient surviving colonies. Colony counts were normalized to the plating efficiency of the 0 Gy control to calculate the surviving fraction. After 10 days, the cells were fixed and stained with crystal violet in 70% ethanol. The number of colonies, defined as clumps of > 50 cells, was counted. The survival fraction was calculated as the ratio of the number of colonies in the treated group to that in the untreated group. Six wells were set up for each condition.

### γH2AX immunohistochemistry staining

For γH2AX immunostaining, after 30 min, 1 h, 4 h, 8 h, and 24 h of radiation exposure, FaDu cells were fixed for 15 min at room temperature with 4% paraformaldehyde and washed twice with PBST (1 × PBS with 0.1% Tween-20). After washing, the cells were incubated with permeabilization buffer (0.3% Triton X-100 in PBS) for 10 min at room temperature and then with blocking buffer (3% bovine serum albumin in PBS) for 1 h at room temperature. After washing, the slides were incubated with primary rabbit anti-γH2AX antibody (Merck KGaA, Darmstadt, Germany) diluted in blocking buffer at 4 °C overnight and then incubated for 30 min at room temperature with an anti-rabbit antibody coupled with fluorescein isothiocyanate (BD, Franklin Lakes, NJ, USA). After washing, the cells were mounted in a medium containing 4′,6-diamidino-2-phenylindole (Life Technologies, Thermo Fisher Scientific, Carlsbad, CA, USA.) to stain their nuclei. Images were automatically acquired using an ImageXpress Micro Confocal System (Molecular Devices, San Jose, CA, USA). At least 250 nuclei were captured, and the number of γH2AX foci/nuclei was determined using MetaXpress software (Molecular Devices).

### Evaluation of the effects of miRNA on the radiotherapy response of a xenograft mouse model

To examine the miRNA’s effects on tumor response to radiotherapy, we subcutaneously injected 8-week-old male mice (BALB/c nu/nu; National Science Council Animal Center, Taipei, Taiwan) with 3 × 10^6^ viable transfected FaDu cells in 100 µL of PBS. After implantation, the xenografts’ tumor volume was measured using calipers according to the formula π/6 × (large diameter) × (small diameter)^2^. Tumor-bearing mice received local irradiation at 13 Gy once their tumor volume reached 150 mm^3^. To assess the tumor’s response to irradiation, we continuously measured the tumor volume to determine its growth rate until it reached 1200 mm^3^. Our previous study described the irradiation protocol in detail [30]. Briefly, tumor-bearing mice were anesthetized with a 1:1 mixture of ketamine (50 mg/kg; Merial Laboratoire de Toulouse, Toulouse, France) and xylazine (20 mg/kg; Bayer HealthCare Animal Health, Leverkusen, Germany) and restrained during irradiation. Their tumors were covered with a 1 cm bolus on the surface and irradiated with 6 MV X-rays from a linear accelerator at a 2–3 Gy/min dose rate. At the experimental endpoint, all animals were humanely euthanized in accordance with protocols approved by the Institutional Animal Care and Use Committee (IACUC, approved number CGU110-071). Mice were first deeply anesthetized via intraperitoneal (IP) injection of a ketamine/xylazine mixture prepared by mixing equal volumes of ketamine (50 mg/mL) and xylazine (1%, Rompun). Each mouse received a total of 80 μL of the anesthetic cocktail (40 μL ketamine + 40 μL Rompun), corresponding to final dosages of 85 mg/kg body weight for ketamine and 15 mg/kg body weight for xylazine. Anesthetic depth was confirmed by the absence of pedal withdrawal and corneal reflexes. Once a surgical plane of anesthesia was achieved, cervical dislocation was performed to ensure rapid and irreversible death. This two-step method was chosen to minimize pain and distress, and is consistent with the AVMA Guidelines for the Euthanasia of Animals (2020).

### Statistical analyses of in vivo and in vitro studies

We used Student’s *t*-test for two-group comparisons in each biological experiment. Results with a *p*-value of < 0.05 were considered statistically significant.

## Results

### Identification of miR-4776-5p as a potential radiosensitizer in head and neck cancer

**Fig. 1:**
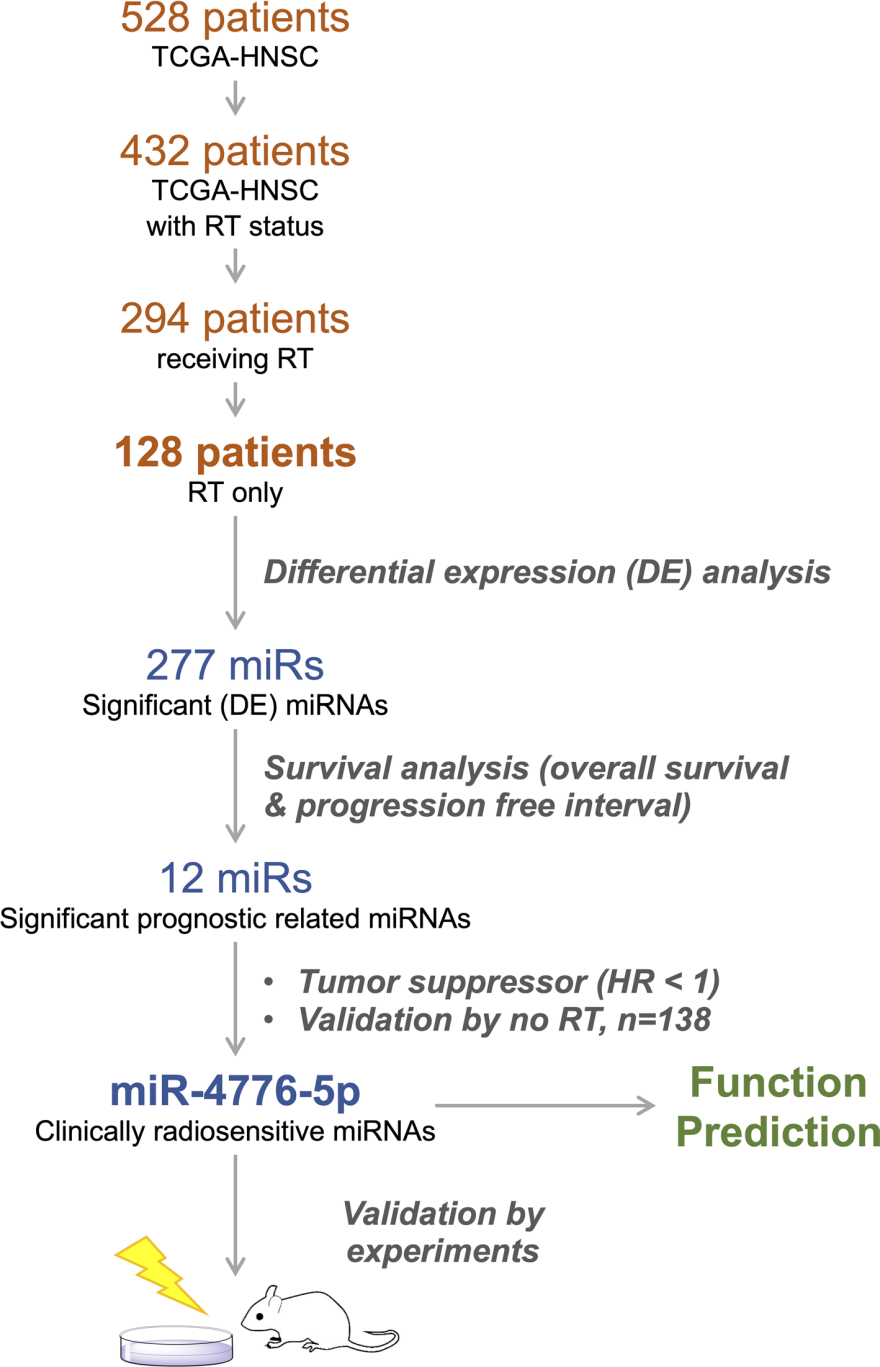
The schematic diagram of the study. RT denotes radiotherapy. “RT only” refers to patients receiving only radiotherapy. "no RT" refers to HNSC patients who did not receive radiotherapy

To identify a candidate miRNA that functions as a radiosensitizer of HNC cells, we employed an integrative bioinformatics approach that combined clinical cohort data with experimental validation (Fig. 1). We first obtained data for 528 patients with HNSC from the TCGA. The radiotherapy status of 432 patients were recorded, with 294 having received radiotherapy. To ensure our focus was on radiosensitizers, we excluded patients who had received other systemic therapies, such as chemotherapy and targeted therapy. This filtering resulted in 128 patients who had received radiotherapy only (Table 1). In addition, 138 patients who did not receive radiotherapy were also identified for comparison (Table S2).

**Table 1:** Basic characteristics of patients receiving radiotherapy alone (RT only). NA indicates that data not available

Characteristic	RT only (N = 128)
Age at diagnosis-yr	
Median (IQR)	64 (55–70)
> = 65 (%)	62 (48.4)
< 65 (%)	66 (51.6)
Sex -no. (%)	
Male	92 (71.9)
Female	36 (28.1)
Stage -no. (%)	
I	5 (3.9)
II	14 (10.9)
III	31 (24.2)
IV	73 (57)
NA	5 (3.9)
Neoplasm histologic grade -no. (%)	
GX	2 (1.6)
G1	17 (13.3)
G2	72 (56.2)
G3	37 (28.9)
Smoking	
Yes	100 (78.1)
No	25 (19.5)
NA	3 (2.3)
Alcohol	
Yes	90 (70.3)
No	34 (26.6)
NA	4 (3.1)

To pinpoint miRNAs associated with radiosensitivity, we conducted a differential expression analysis comparing primary tumors of the 128 radiotherapy-only patients and their adjacent normal tissue. This analysis revealed 277 miRNAs that were significantly differentially expressed between the tumor and normal tissue (Figure S1). Further survival analysis, using overall survival and progression-free interval as endpoints, identified 12 miRNAs that were significantly associated with disease progression in HNSC patients who received radiotherapy only (Figure S2). Importantly, these miRNAs were not linked to clinical outcomes in the 138 patients who did not receive radiotherapy (Figure S3), suggesting a specific association with the radiotherapy response.

**Fig. 2:**
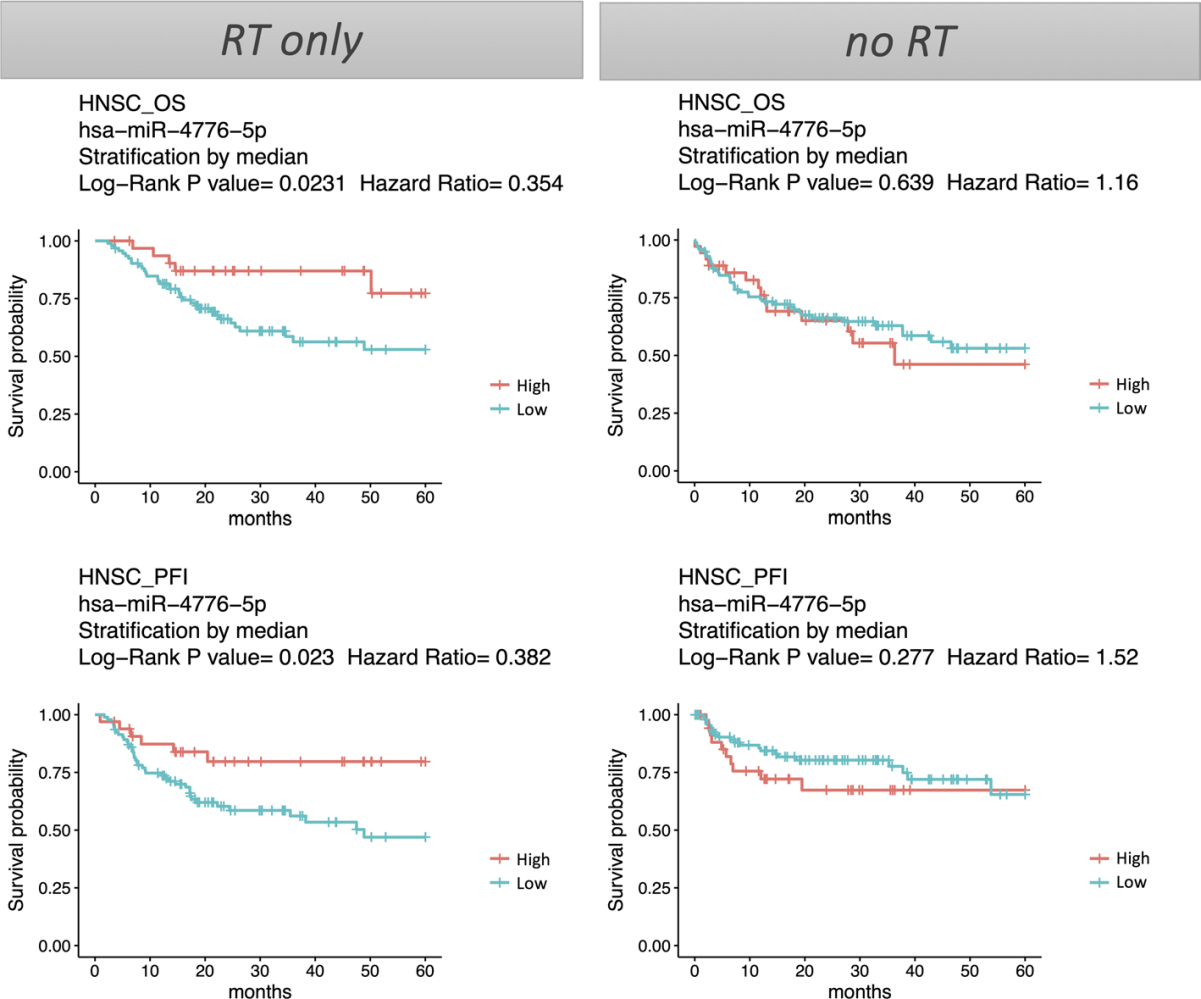
Survival analysis of HNC patients stratified by miR-4776-5p. Kaplan–Meier survival plot of overall survival (OS) and progression-free interval (PFI) in HNC patients receiving radiotherapy only (RT only) or not receiving radiotherapy (no RT)

Among the 12 candidate miRNAs, miR-4776-5p stood out as the only one whose high expression was associated with better prognosis (hazard ratio < 1) in the radiotherapy-treated HNSC group but not in the non-radiotherapy group (Fig. 2). This finding suggests that the prognostic role of miR-4776-5p is likely due to its impact on radiosensitivity rather than intrinsic tumor behavior (Fig. 2). Consequently, miR-4776-5p emerged as a promising radiosensitizer for potential drug development and as a biomarker for stratifying HNC patients for radiotherapy. Based on these insights, we selected miR-4776-5p for further detailed analysis and experimental validation.

### miR-4776-5p is associated with the cell cycle and immune response

To elucidate the mechanism by which miR-4776-5p functions as a radiosensitizer in HNC, we performed a systematic analysis to identify its target genes and their associated biological functions. Using an array of 12 bioinformatics tools, we predicted the putative target genes of miR-4776-5p. We then focused on those genes whose expression levels were negatively correlated with miR-4776-5p expression, specifically in the 128 patients who had received only radiotherapy. These negatively correlated genes were identified as potential direct targets of miR-4776-5p.

Subsequent functional enrichment analysis of these target genes revealed significant involvement in pathways related to the cell cycle and immune response, both of which are crucial for modulating radiosensitivity (Table S2). The involvement of these pathways suggests that miR-4776-5p may enhance radiosensitivity by interfering with cell cycle regulation and immune-mediated tumor responses, which are key factors of effectiveness of radiotherapy.

To further validate these findings, we randomly selected six of the identified target genes for qRT-PCR validation. The results confirmed that the expression of these six genes was significantly suppressed following transfection with miR-4776-5p mimics (Figure S4), supporting the hypothesis that miR-4776-5p directly downregulated genes involved in pathways critical to radiosensitivity. This experimental validation underscores the potential of miR-4776-5p as a key regulator of the radiotherapy response of HNC.

### MiR-4776-5p suppresses DNA repair and sensitizes HNSC cells to ionizing radiation

**Fig. 3:**
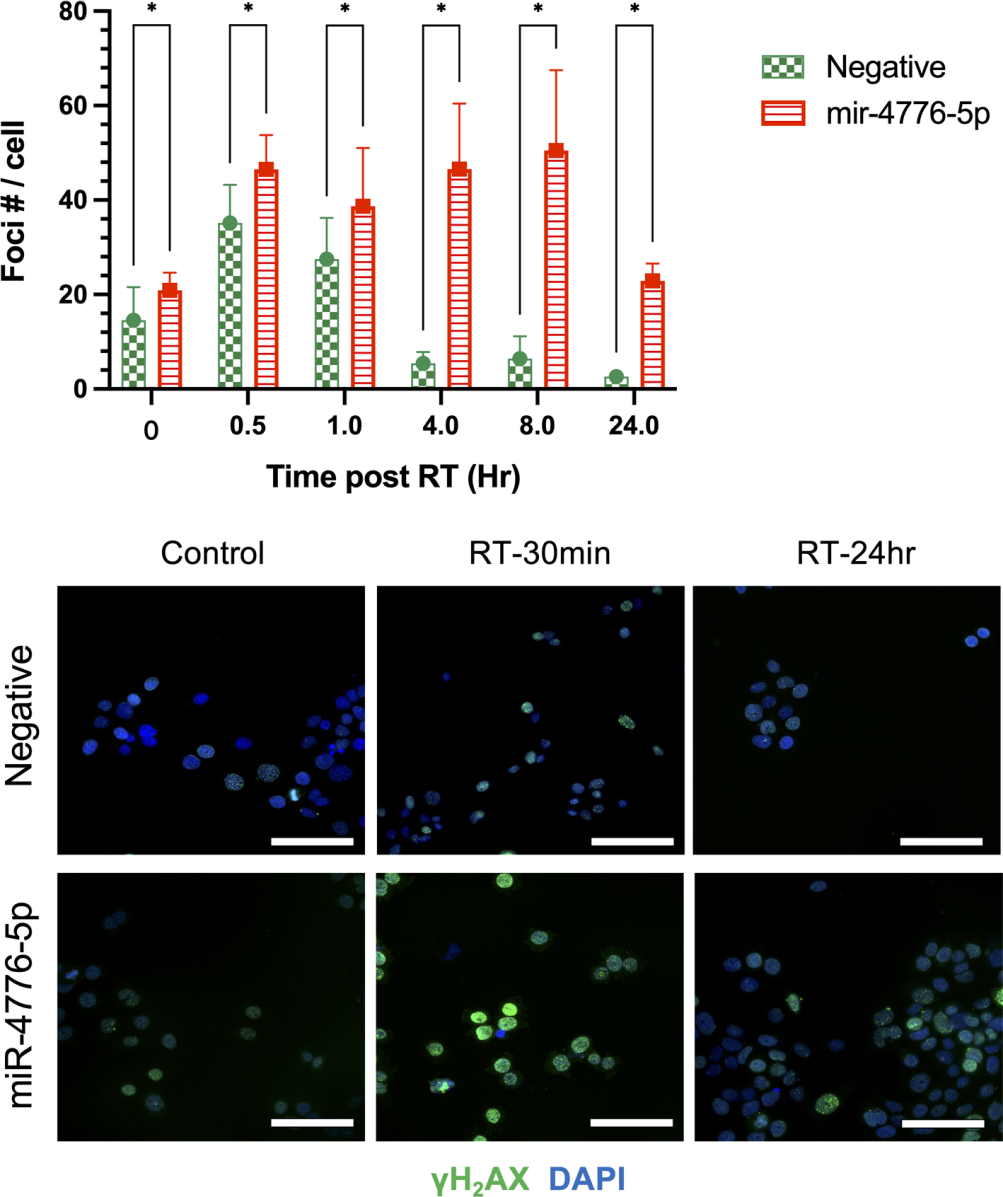
The effect of miR-4776-5p overexpression on DNA repair capacity in Fadu cells. The upper panel shows the quantification of DNA double-strand breaks (DSBs) induced by 2 Gy irradiation, measured by the number of γH2AX foci in cells with or without miR-4776-5p overexpression. Statistical significance is indicated by *, p < 0.0001. The lower panel displays representative immunofluorescent images of γH2AX foci (green) in the nuclei (blue) of the cells. Scale bar: 100 µm

To investigate the impact of miR-4776-5p on DNA damage and repair efficiency in FaDu cells, we examined the extent of DNA double-strand breaks (DSBs) induced by radiation. H2AX phosphorylation, indicated by the presence of γH2AX foci, serves as a reliable marker for assessing DSB formation and repair kinetics. We quantified γH2AX foci at various time points following radiation in FaDu cells transfected with either miR-4776-5p mimics or negative control (NC) miRNAs. As shown in Fig. 3, 2 Gy of radiation caused a significant increase in γH2AX foci in both miR-4776-5p and NC transfected cells 30 min after radiation. However, in cells transfected with miR-4776-5p, the γH2AX foci persisted up to eight hours after radiation, indicating a delay in DNA repair compared to the NC group. This suggests that miR-4776-5p may impair the DNA repair machinery, leading to prolonged DNA damage.

**Fig. 4:**
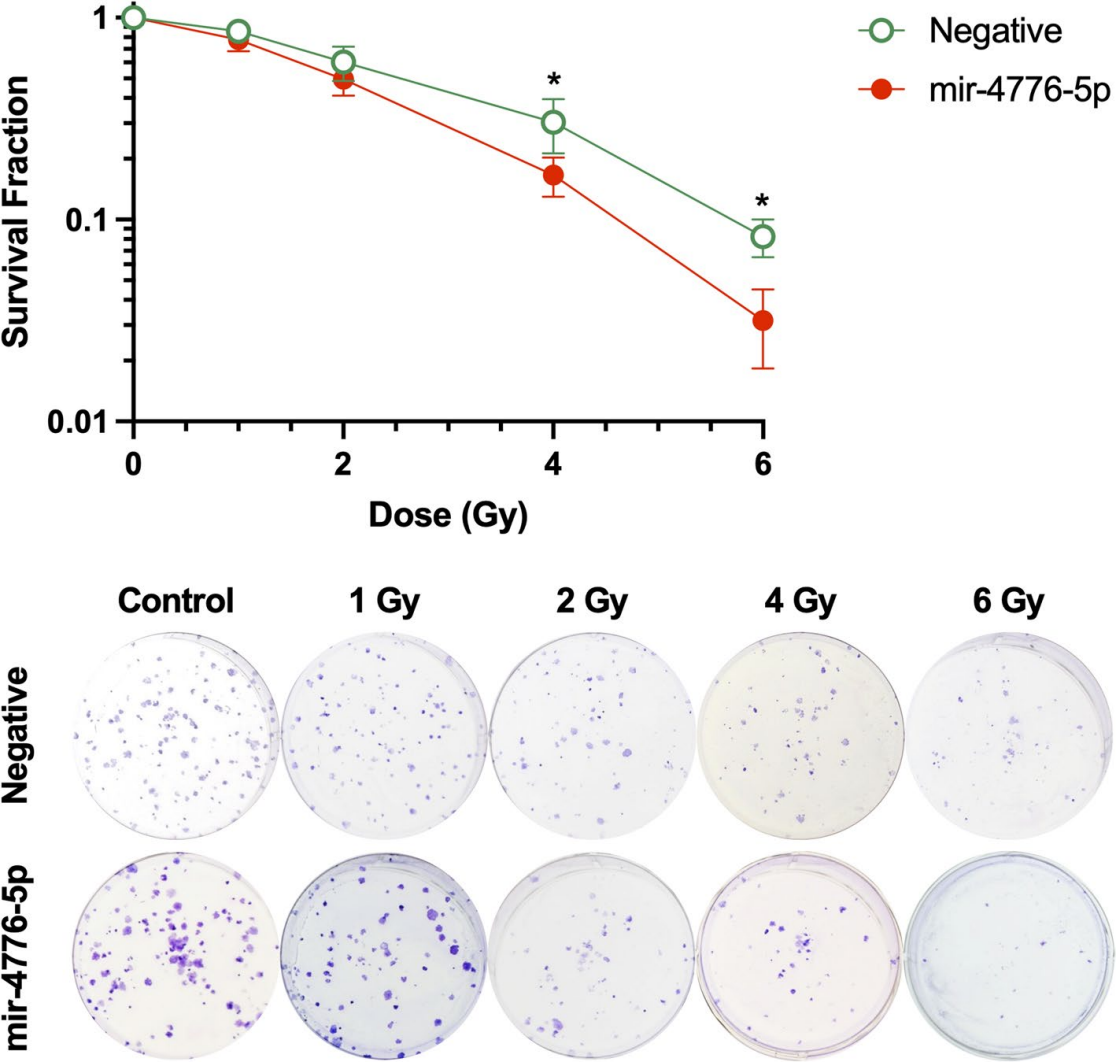
The impact of miR-4776-5p overexpression on the radiosensitivity of Fadu cells. The upper panel shows the analysis of surviving fractions of Fadu cells exposed to increasing doses of Cs-137 radiation, with or without miR-4776-5p overexpression. Statistical significance is indicated by *, p < 0.001. The lower panel displays representative images of colony morphology and numbers for Fadu cells subjected to varying doses of Cs-137 radiation, comparing cells with miR-4776-5p overexpression to those without

Furthermore, we assessed the effect of miR-4776-5p overexpression on radiosensitivity using a clonogenic assay. FaDu cells transfected with miR-4776-5p or NC miRNAs were exposed to increasing doses of radiation, and their survival was monitored. As illustrated in Fig. 4, no significant differences were observed at the lower radiation doses (0–2 Gy). In contrast, at 4 Gy and 6 Gy, the survival fraction of the miR-4776-5p overexpression group was significantly lower than that of the negative control group (p < 0.05). These findings indicate that miR-4776-5p enhanced the radiosensitivity of FaDu cells, possibly through the persistence of DNA damage and a corresponding decrease in cell viability following radiation exposure.

### miR-4776-5p increases tumor radiosensitivity and delays tumor growth in HNSC xenografts

**Fig. 5:**
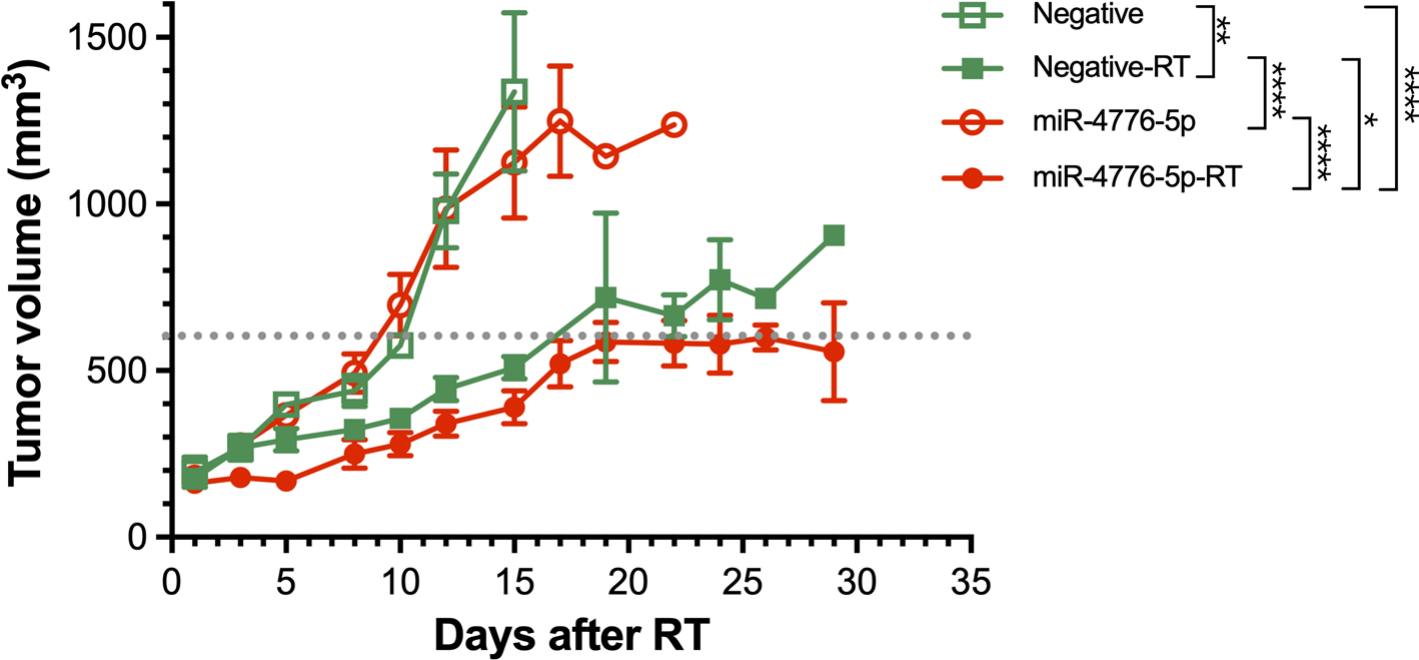
miR-4776-5p enhanced RT’s tumor suppression capability in vivo. Tumor growth curves of xenografts after a single 13 Gy irradiation. miR-4776-5p transfection alone did not significantly alter tumor growth compared with the negative control. In combination with radiotherapy, miR-4776-5p significantly delayed tumor progression compared with controls (*p < 0.05, **p < 0.01, ***p < 0.001). Data are shown as mean ± SD

Increasing the levels of miR-4776-5p in FaDu cells significantly suppressed DNA DSB repair and induced mitotic cell death. To evaluate whether this effect translated to enhanced radiosensitivity in vivo, we conducted experiments using an immune-deficient BALB/C nude mouse model. FaDu cells transfected with either miR-4776-5p mimics or a scramble NC were inoculated into the mice, and tumors were allowed to grow until they reached a volume of 150 mm^3^. At this point, the tumors were subjected to a single 13 Gy dose of ionizing radiation. Statistical analysis showed that miR-4776-5p transfection alone did not significantly affect the intrinsic tumor growth rate, as both the miR-4776-5p and negative control groups reached ~ 600 mm^3^ by day 9 (ns). Following irradiation, however, tumor growth was significantly delayed in the miR-4776-5p group compared to controls (Fig. 5). Tumors in the miR-4776-5p + RT group required 19 days to reach 600 mm^3^ and remained stably suppressed up to day 29, whereas tumors in the NC + RT group reached the same size by day 17 (p < 0.05). Compared with their respective non-irradiated controls, radiotherapy induced an average tumor growth delay of ~ 8 days in the NC group and ~ 20 days in the miR-4776-5p group. These differences were statistically significant. These findings further corroborate that miR-4776-5p overexpression enhanced the radiosensitivity of HNSC cells, effectively slowing tumor growth after ionizing radiation.

## Discussion

Radiotherapy plays a crucial role in the definitive and adjuvant treatment of HNC; however, radioresistance remains a significant obstacle to successful treatment outcomes. This study addressed this critical issue by applying an integrative bioinformatics approach to the investigation of miR-4776-5p, a microRNA that regulates tumor radiosensitivity both in vitro and in vivo. It found that miR-4776-5p not only influenced the response of HNC cells to radiation, but also served as a predictor of prognosis in patients undergoing radiotherapy. The ability of miR-4776-5p to enhance radiosensitivity indicates its potential as a prognostic biomarker for stratifying patients who would benefit the most from radiotherapy. Moreover, miR-4776-5p could serve as a potential therapeutic target for the development of novel radiosensitizers, offering new avenues to overcome radioresistance and improve treatment efficacy in HNC.

While numerous studies have explored the role of miRNAs in modulating the radiosensitivity of HNCs, few have demonstrated consistency in results between bench experiments and clinical outcomes [1]. Many previous investigations focused on analyzing changes in miRNA expression in HNC cell lines in vitro, initially identifying candidates through microarray detection. However, only a limited number of these studies validated their findings with clinical samples [10, 19]. With the advent of high-throughput next-generation sequencing technologies, which offer greater sensitivity than microarray methods, large sequencing databases such as TCGA have become invaluable resources for research. Despite this, only two studies to date have used these big data resources to explore miRNAs related to radiosensitivity in HNC patients through integrative bioinformatics approaches [4, 13]. One study proposed a nomogram based on five miRNA signatures to predict the radiotherapy response of HNSC patients, while relying solely on in silico data analysis [4]. The other study identified miR-130b as a potential biomarker of radiosensitivity in HPV-negative oropharyngeal squamous cell carcinoma using TCGA data [13]. However, both studies had limitations, particularly in distinguishing the effects of radiotherapy from those of other therapies, such as chemotherapy and targeted treatments, and in the lack of bench validation to confirm the role of miRNA in radiosensitivity.

In contrast to these previous studies that primarily focused on in silico analysis for identifying cancer-related miRNA biomarkers, our approach involved a more rigorous selection process. We carefully curated a cohort of HNC patients who received only radiotherapy and compared them with a group of patients who did not receive radiotherapy. This dual-group analysis allowed us to identify miRNAs that were significant in the radiotherapy-only group but not in the non-radiotherapy group, providing a clear indication that these miRNAs were specifically related to radiosensitivity rather than to general tumor progression. By validating our candidate miRNAs in a clinical cohort of HNSC patients not receiving radiotherapy, we further strengthened the evidence that miR-4776-5p plays a crucial role in regulating the radiotherapy response. The clear inconsistency between the radiotherapy-only and non-radiotherapy groups suggests that miR-4776-5p is involved in modulating radiosensitivity rather than merely affecting HNC progression. Through this strategy, we were able to identify miR-4776-5p as a promising radiosensitizer from an initial list of 12 candidate miRNAs and highlight its potential as both a prognostic biomarker and a therapeutic target in HNC treatment.

miR-4776-5p is a relatively recently discovered miRNA, with limited literature available on its functional roles. Based on miRTARBase [8], which provides the experimental evidence of miRNA-gene interaction, there are only 30 genes that can be regulated by miR-4776-5p. However, there are only 2 genes passed our correlation analysis (Table S1). Across TCGA samples, miR-4776-5p expression is reduced in tumor relative to adjacent nor-mal tissue, consistent with tumor-suppressive down-regulation. Normal tissues showed no difference by RT status, as expected for baseline surgical specimens, and tumor expression was broadly similar in patients who did versus did not receive RT (Figure S5). Despite overall down-regulation in cancer, patients whose tumors retain relatively higher miR-4776-5p exhibit superior survival. Our study shows miR-4776-5p has a tumor-suppressive and potentially radiosensitizing role in head and neck cancer.

The functional enrichment results for the putative target genes of miR-4776-5p (see Table S4) suggest that the mechanisms of this miRNA may be related to cell cycle regulation and immune response, implying a connection to radiosensitivity. However, these findings are preliminary and require further experimental validation. Additionally, a limited number of existing studies indicate that miR-4776-5p plays a role in key regulatory pathways, particularly in the context of cancer. For instance, miR-4776-5p has been shown to be involved in the regulation of NF-κB, with upregulation correlated with a decrease in NF-κB inhibitor beta (NFKBIB) expression [24]. This finding aligns with our predictive analysis, which indicated that miR-4776-5p might play a role in immune responses and cell cycle regulation. Moreover, miR-4776-5p has been reported in an miRNA–gene regulatory network for gastric cancer [31], implying that it might have broader implications for oncogenesis. Our analysis further supported these functions, suggesting that miR-4776-5p could be involved in the DNA damage response and apoptosis—mechanisms traditionally associated with radiation-induced tumor inhibition. In addition, recent studies highlight the potential immunomodulatory effects of radiation, such as the abscopal effect, in which local tumor irradiation also inhibits distant metastatic lesions [12, 15, 25]. These insights reinforce the significance of miR-4776-5p in modulating both the direct and indirect effects of radiotherapy.

Our experimental validation strongly supports the predicted roles of miR-4776-5p. In particular, our data demonstrated that miR-4776-5p significantly impaired DNA repair processes, as evidenced by the quantification of γH2AX foci, where irradiated cells overexpressing miR-4776-5p showed delayed resolution of DSBs. This delay in DSB repair likely contributes to the reduced survival of HNSC cells following radiotherapy, as unrepaired DNA damage leads to failed cell division and increased cell death. Furthermore, our in vivo studies revealed that while miR-4776-5p overexpression did not influence tumorigenesis, it significantly enhanced tumor radiosensitivity, as indicated by the prolonged inhibition of growth of miR-4776-5p-transfected tumors after irradiation. In our qRT-PCR validation (Figure S4), the changes in expression of six potential target genes did not reach statistical significance (p > 0.05) after transfection with miR-4776-5p mimics. While individual comparisons did not meet the predetermined significance threshold, we observed that all six genes demonstrated consistent downregulation following miRNA mimic transfection. Assuming a simple null hypothesis where upregulation or downregulation is equally likely for each gene, the probability of observing all six genes showing changes in the same direction is 1 in 64, or approximately 1.6%. This suggests that the overall pattern we see is unlikely to be purely random. We present this consistency as supportive, hypothesis-generating evidence, while being careful not to over-interpret the results for any single gene.

Our research suggests that miR-4776-5p may serve as a radiosensitizer, but immediate clinical application is limited by several factors. First, our preclinical validation used a single head and neck squamous cell carcinoma (HNSC) model in immunodeficient mice. To enhance general applicability, further testing across diverse and HPV-stratified HNSC models is needed, along with assessments of potential effects on normal tissues. Second, while microRNAs can regulate various pathways, this can lead to off-target effects due to partial complementarity, potentially causing unintended harm in specific tissues. Thus, comprehensive profiling for these effects, along with dose–response assessments, is necessary. Third, translating miRNA-based radiosensitizers into clinical practice faces challenges such as delivery, biodistribution, and immunogenicity. Efforts are underway to optimize chemistry and improve delivery methods to ensure selective tumor uptake while minimizing exposure to normal tissues. Finally, our retrospective cohort analysis, although carefully stratified by treatment type, is observational. Prospective validation in independent cohorts and studies in pharmacology and toxicology will be the next crucial steps.

## Conclusion

Our integrative analysis of the clinical cohorts—explicitly contrasting patients who received radiotherapy alone with those who received no radiotherapy—together with mechanistic in vitro and in vivo experiments, nominates miR-4776-5p as a candidate radiosensitizer and a prognostic marker for patients undergoing radiotherapy for head-and-neck cancer. Functionally, enforced miR-4776-5p expression delayed DNA double-strand break repair and reduced clonogenic survival, and, in xenografts, augmented radiation-induced tumor growth delay. These convergent results support the biological plausibility that miR-4776-5p can modulate pathways relevant to radiosensitivity and motivate further translational exploration.

## Supplementary Information

Supplementary file 1.

Supplementary file 2.

## Data Availability

The datasets analyzed in this study are publicly available from The Cancer Genome Atlas (TCGA) – Head and Neck Squamous Cell Carcinoma (TCGA-HNSC) project via the Genomic Data Commons (GDC; (Project accession: TCGA-HNSC). The miRNA-seq data were originally downloaded from CGHub and processed using the pipeline provided by YM500. The RNA-seq expression profiles and clinical information were accessed through DriverDB, which retrieves harmonized TCGA datasets from GDC. All other data supporting the findings of this study are available within the article and its supplementary materials, or upon reasonable request from the corresponding authors.
