# Supplementary material for: Integrative multi-omics and preclinical analyses identify miR-4776-5p as a prognostic radiosensitizer for patients undergoing radiotherapy for head-and-neck cancer
Source: Discov Oncol. 2025 Oct 28;16:1979. doi: 10.1007/s12672-025-03784-6 (PMC12569267; doi:10.1007/s12672-025-03784-6)
Supplement: Supplementary file 1 [file 12672_2025_3784_MOESM1_ESM.docx]

# Supplementary Figure

**Figure S1**: The heatmap illustrates the differentially expressed miRNAs that can differentiate between the primary tumors and the adjacent normal tissues.


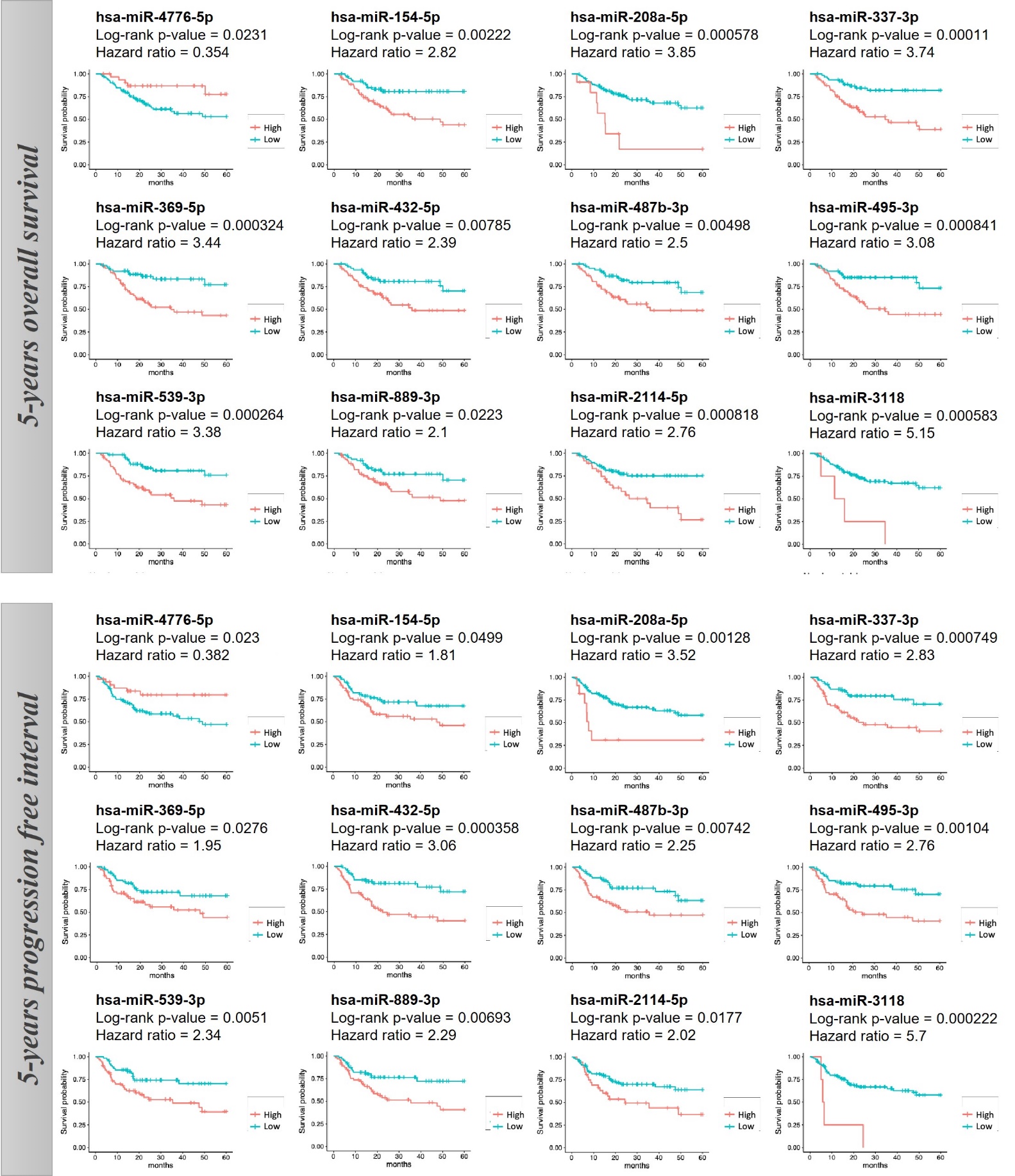


**Figure S2**: Survival analysis of the 12 candidate miRNAs in patients undergoing radiotherapy only (RT only).


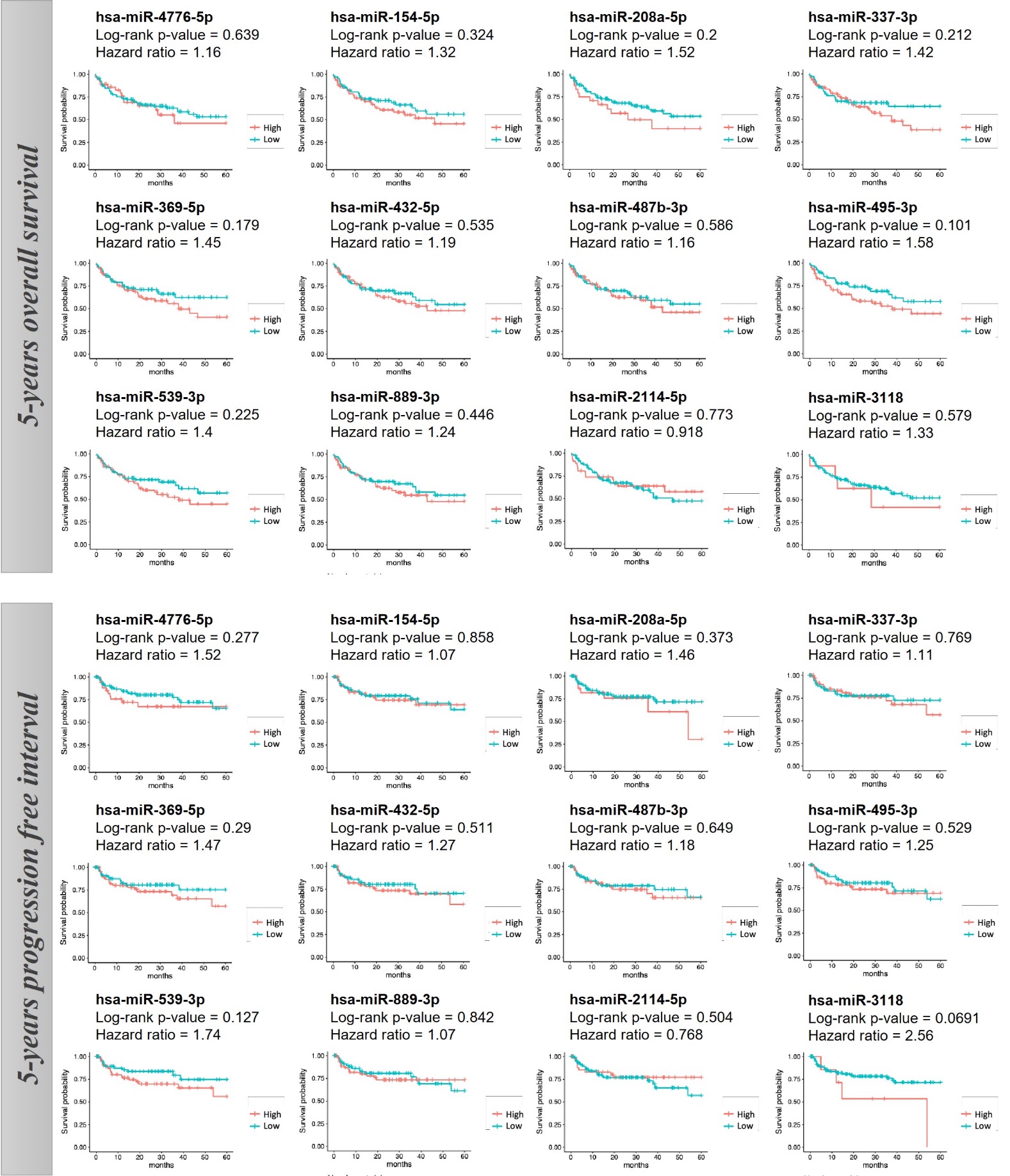


**Figure S3:** Survival analysis of the 12 candidate miRNAs in patients who did not receive radiotherapy (no RT).


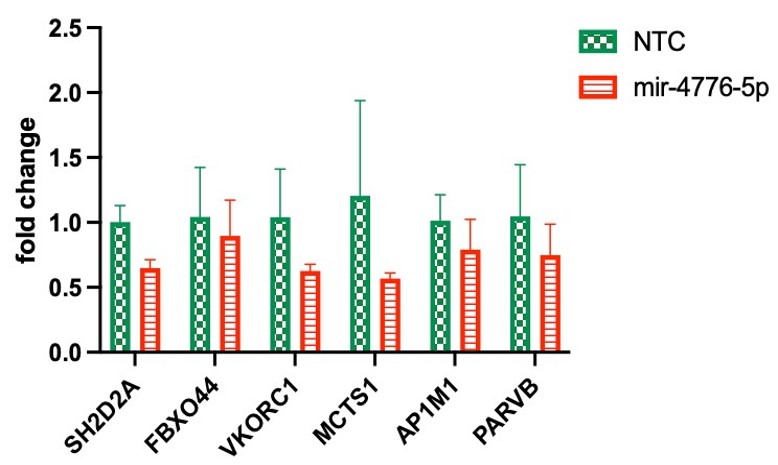


**Figure S4**: The qRT-PCR validation of the six randomly-selected target genes.

**Figure S5:** The Expression level of miR-4776-5p in adjacent normal (NP) and tumor (TP) tissues stratified by radiotherapy (RT) status.
